# Crop filling: A pipeline for repairing memory clinic MRI corrupted by partial brain coverage

**DOI:** 10.1016/j.mex.2023.102542

**Published:** 2024-01-11

**Authors:** Gonzalo Castro Leal, Tim Whitfield, Janaki Praharaju, Zuzana Walker, Neil P. Oxtoby

**Affiliations:** a Department of Computer Science, UCL Centre for Medical Image Computing, University College London, London, UK; b Division of Psychiatry, University College London, London, UK; c Princess Alexandra Hospital NHS Trust, Essex, UK; d Essex Partnership University NHS Foundation Trust, Essex, UK

**Keywords:** Magnetic resonance image, Artefacts, Real world data, MRI Crop Filling

## Abstract

Data-driven solutions offer great promise for improving healthcare. However, standard clinical neuroimaging data is subject to real-world imaging artefacts that can render the data unusable for computational research and quantitative neuroradiology. T1 weighted structural MRI is used in dementia research to obtain volumetric measurements from cortical and subcortical brain regions. However, clinical radiologists often prioritise T2 weighted or FLAIR scans for visual assessment. As such, T1 weighted scans are often acquired but may not be a priority, resulting in artefacts such as partial brain coverage being systematically present in memory clinic data.

Here we present “MRI Crop Filling”, a pipeline to replace the missing T1 data with synthetic data generated from the T2 scan, making real-world clinical T1 data usable for computational research including the latest AI innovations. Our method consists of the following steps:

• Register scans: T2 and (cropped) T1.
• Synthesise a new T1 using an open source deep learning tool.
• Replace missing (cropped) T1 data in original T1 scan and super-resolve to improve image quality.

## Graphical abstract

**Figure:**
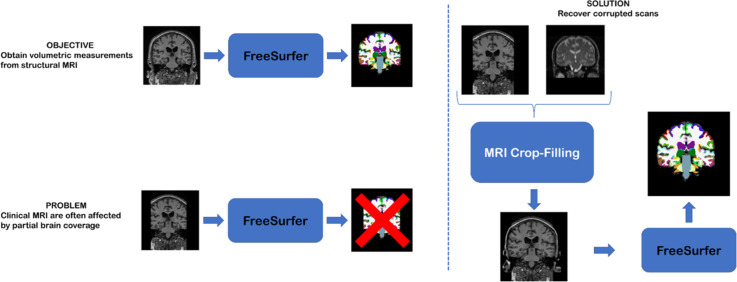

Specifications table

**Table:** 

Subject area:	Neuroscience
More specific subject area:	Neuroimaging
Name of your method:	MRI Crop Filling
Name and reference of original method:	N/A
Resource availability	https://github.com/ucl-codec/mri_crop_filling

**Method details**

## Method description

Our new method is based on two existing tools in FreeSurfer (a popular neuroimaging analysis software — see Supplementary Material), and requires a T1-weighted and a T2-weighted MRI scan for each patient, both acquired on the same day. The tools are *SynthSR* and *SynthSR Hyperfine* [[1], [2]], which generate a synthetic T1w contrast scan with 1 × 1 × 1 mm voxel size using a T2w scan. We have containerised these FreeSurfer functionalities for the benefit of the community. The docker file and details on how to build the image are further explained in our github repository linked above.

1. Zeropad the partial coverage T1w scan.
2. It is important that the field of view (FOV) of both T1w and T2w scans cover the same volume.
3. Register T2w to T1w.
  Registering the T2w scan to the T1w scan space is mandatory for the use of *SynthSR Hyperfine*, but also ensures that synthetic scans obtained from the T2w scan will already be registered. Here this registration is performed using Free-Surfer tool *mri_robust_register* [3] following the recommendations made by the *synthSR* developers (https://surfer.nmr.mgh.harvard.edu/fswiki/SynthSR).
4. Create the first synthetic T1w.
  The registered T2w scan is used to obtain a synthetic T1w using the *SynthSR* tool. The output is a synthetic T1w scan of 1 mm^3^ isotropic voxel dimension and 256 × 256 × 256 mm^3^ FOV with contrast resembling an MPRAGE (*Magnetization Prepared RApid Gradient Echo*) acquisition.
5. Interpolating the synthetic T1w scan.
  The voxel and FOV dimensions both need to match the scan to be filled. First, we use the ‘mri_convert’ tool from FreeSurfer to ensure that the voxels of both scans sample the same physical space when performing interpolation. Then, we trim any extraneous voxels from the synthetic T1w image (most original T1w images will not have the uniform grid of 256 × 256 × 256 voxels as produced in the preceding step by SynthSR). This ensures that the T1w image matrices (original and synthetic) are the same size.
6. Obtain the first filled scan (Fig. 1).

**Fig. 1:**
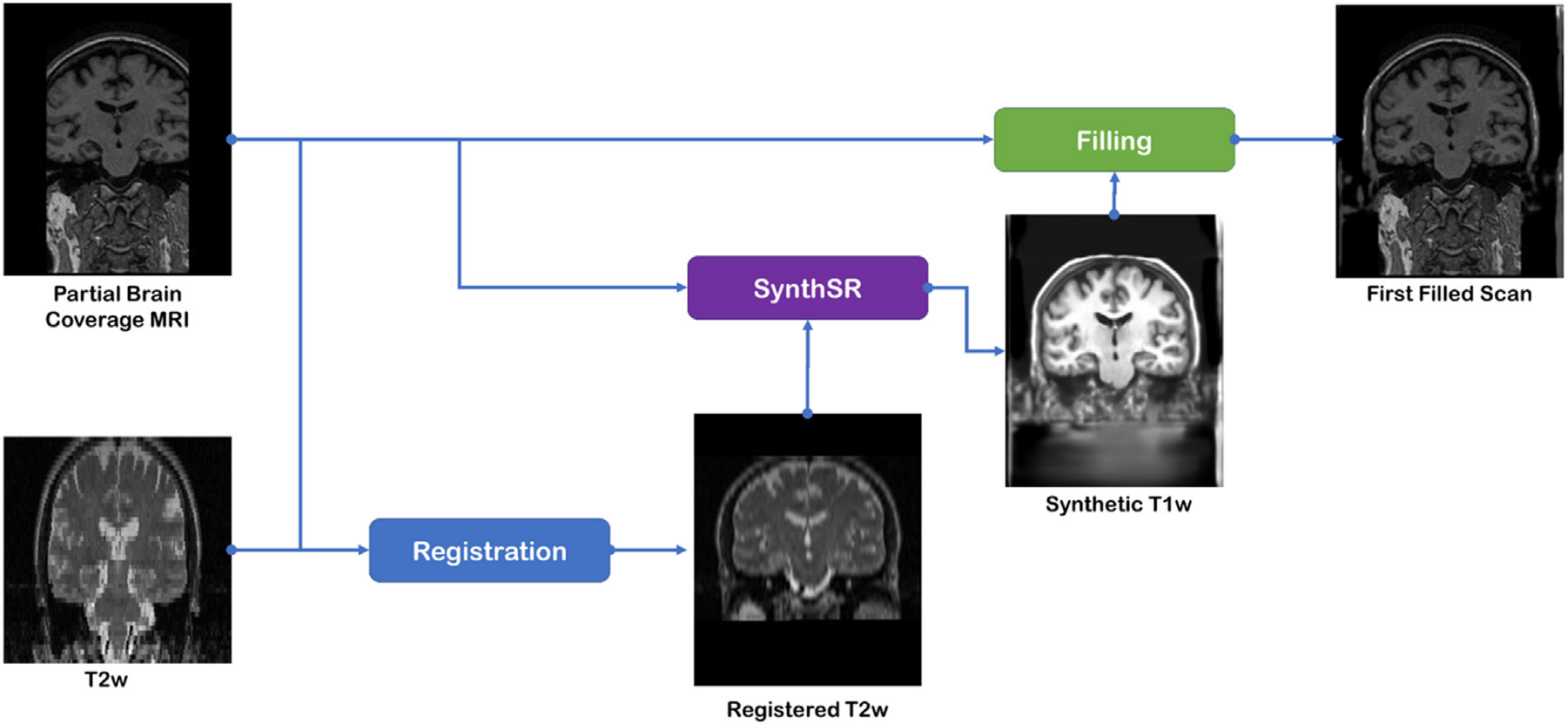
Flow diagram of the ‘MRI Crop-Filling’ pipeline steps 1–5, producing the first filled T1w scan.
  The zeroed voxels corresponding to missing data in the original T1w scan due to partial brain coverage are replaced by the values in the interpolated synthetic T1w scan.
7. Create the second synthetic T1w (Fig. 2).

**Fig. 2:**
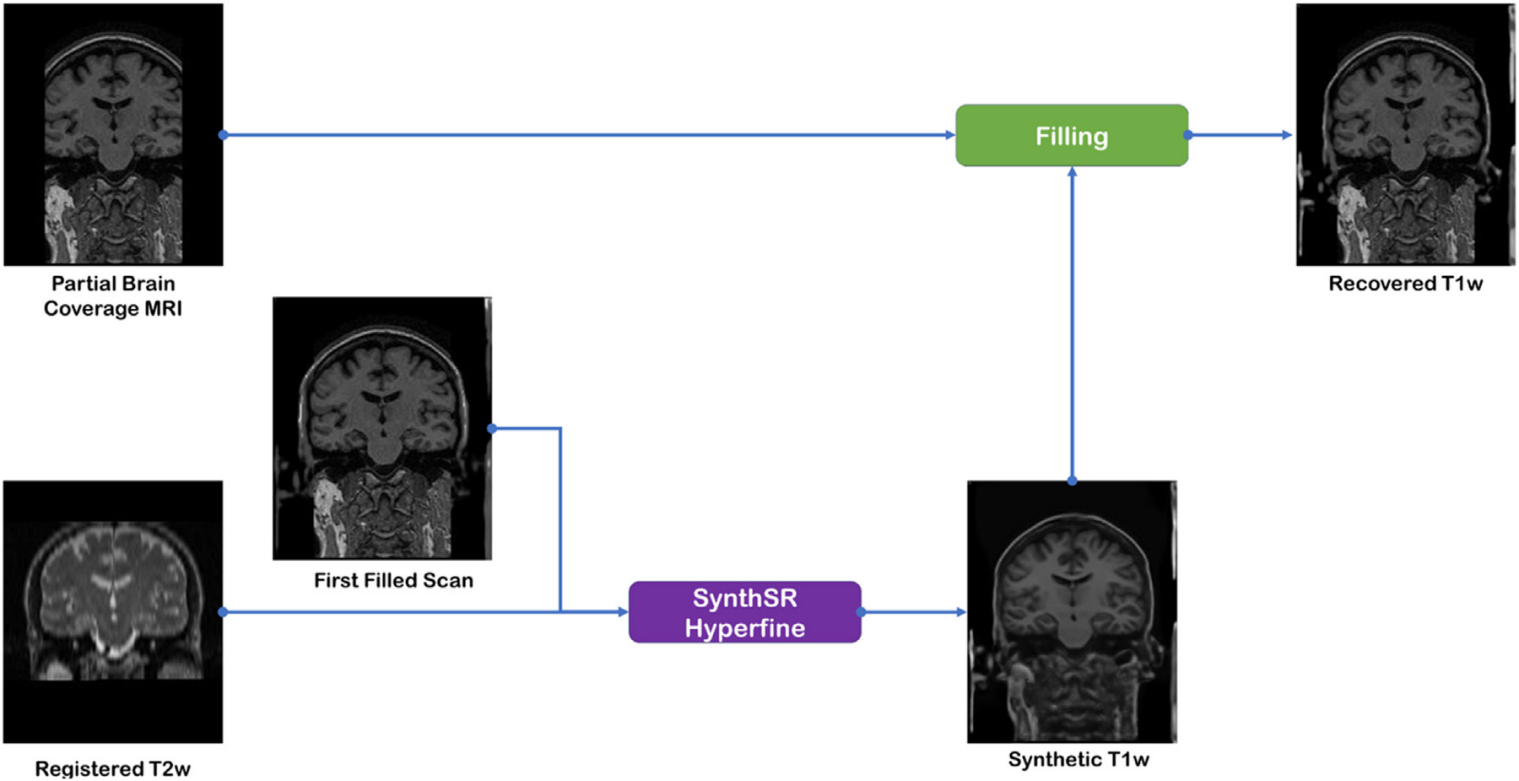
Flow diagram of the ‘MRI Crop-Filling’ pipeline final steps necessary to obtain the fully repaired T1w MRI scan.
  The filled T1w scan is now used in combination with the registered T2w scan, using *SynthSR Hyperfine tool,* to obtain a more accurate synthetic T1w scan. Finally, repeat steps 4 and 5 with the new synthetic scan to produce the final result.

## Method validation

**Fig. 3:**
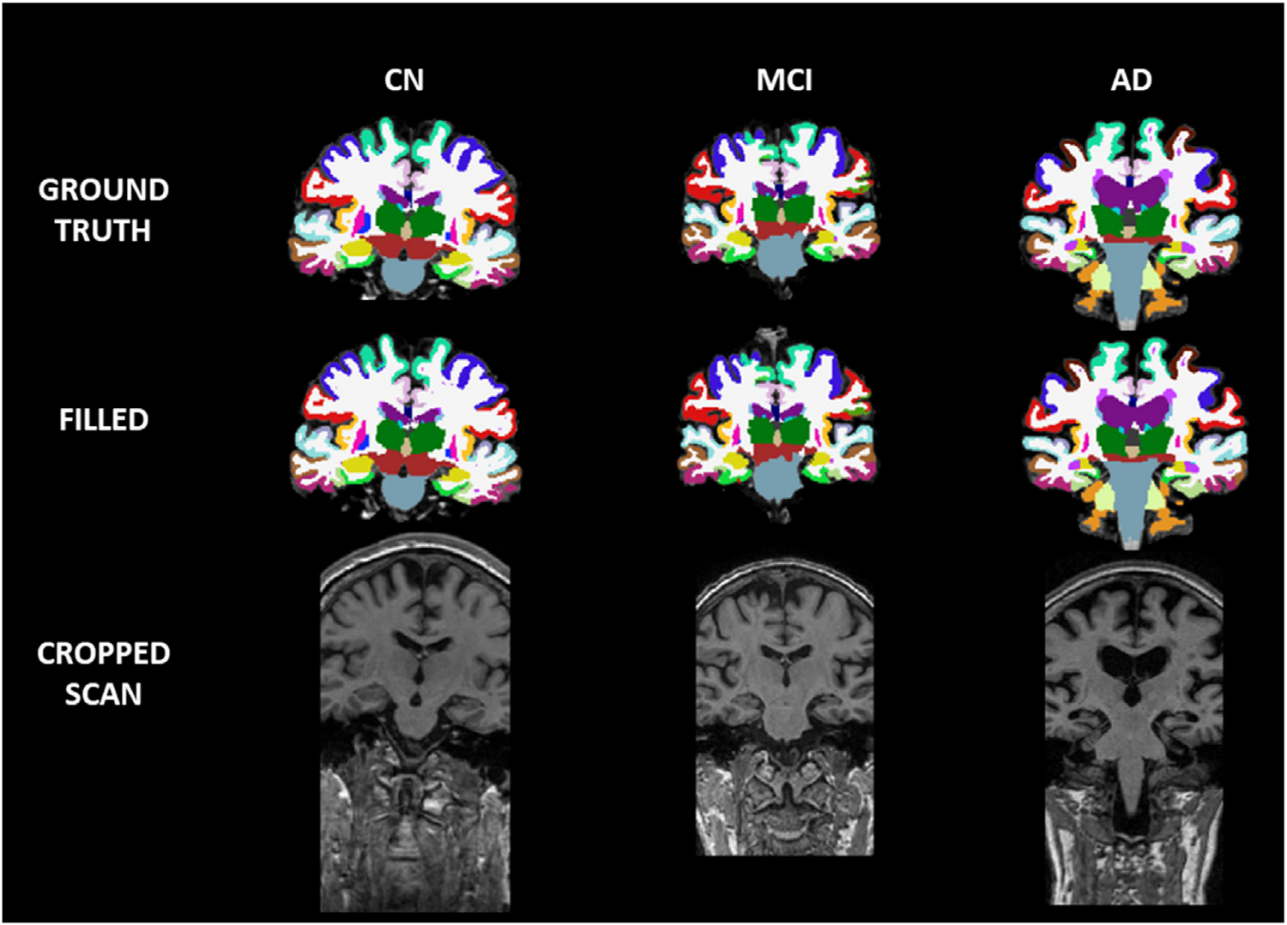
Segmentation brain labels for CN, MCI and AD participants of ground truth and filled scans. The bottom row depicts the severity of the partial volume coverage seen in the axial plane of the T1w MRI scans.

Examples of results for the segmentation of filled scans with the developed pipeline are presented in Fig. 3, where they are displayed against the ground truth labels and the cropped scan they were recovered from. For details on the dataset used to assess the validation of our methods, see Supplementary Material / Additional Information. The artefact was induced manually on a cohort of scans, which were later analysed with FreeSurfer (before the corruption, artefact-corrupted, and after repair). Performance of the method was evaluated based on correlation and bias metrics, with statistical tests to estimate the disease signal recovery and in clinical classification tasks relevant to Alzheimer's disease progression. For details on how these metrics were calculated, see Additional Information.

## Ethics statements

Our secondary analysis of data from the ADNI and CODEC studies has been approved by UCL's Research Ethics Committee (UCL/REC 8019/005). All participants in both studies provided informed consent.

## CRediT authorship contribution statement

**Gonzalo Castro Leal:** Conceptualization, Data curation, Formal analysis, Methodology, Software, Writing – original draft, Writing – review & editing. **Tim Whitfield:** Writing – review & editing. **Janaki Praharaju:** Data curation, Writing – review & editing. **Zuzana Walker:** Funding acquisition. **Neil P. Oxtoby:** Conceptualization, Funding acquisition, Methodology, Data curation, Supervision, Writing – original draft, Writing – review & editing.

## Declaration of competing interest

The authors declare that they have no known competing financial interests or personal relationships that could have appeared to influence the work reported in this paper.

## Data Availability

ADNI data is publicly available. Code is available on GitHub.

## Appendix Supplementary materials

Image, application 1
