# Supplementary material for: Crop filling: A pipeline for repairing memory clinic MRI corrupted by partial brain coverage
Source: MethodsX. 2024 Jan 11;12:102542. doi: 10.1016/j.mex.2023.102542 (PMC10837087; doi:10.1016/j.mex.2023.102542)
Supplement: Supplementary file 1 [file mmc1.docx]

**Supplementary material *and/or* additional information**

#### Background

Semi-automated tools for brain segmentation have empowered large scale quantitative analysis of regional neurodegeneration in MRI scans. Popular software includes FreeSurfer (<https://surfer.nmr.mgh.harvard.edu/>) and FSL (Analysis Group, FMRIB, Oxford, UK; <https://fsl.fmrib.ox.ac.uk/fsl/fslwiki>). Such brain segmentation tools typically use whole brain atlases or templates. For example, FreeSurfer uses brain topology to establish cortical boundaries (4) and FSL-FIRST relies on a whole-head registration to the MNI152 atlas (5). These tools have been used extensively to study AD using regional brain volumes, e.g., for disease clustering and prognosis (6–8), diagnostic utility (9–12), and atrophy rates during disease progression (13,14).

Neuroimage data used in semi-automated brain segmentation tools should be carefully selected, as multiple factors can affect accuracy and reliability of results. Quality controls include assessment of technical artefacts such as head coverage, radiofrequency noise, signal inhomogeneity and susceptibility, and motion artefacts. Such technical artefacts are more common in real-world data than in the highly controlled clinical research studies typically leveraged by researchers to develop new quantitative methods for neuroimage analysis. Among these factors, partial brain coverage was by far the most prevalent one encountered in the CODEC dataset of routinely collected data from the Essex Memory Clinic near London in the United Kingdom (15). Investigation revealed that this resulted from an operational decision to reduce patient time in the MRI scanner, with T2w MRI prioritised for visual assessment and T1w MRI scans cut short.

##### Study Design and Participants

Data was selected from the Alzheimer’s Disease Neuroimaging Initiative (ADNI; https://adni.loni.usc.edu/) observational research study. Data from the real-world CODEC memory clinic study was used to inform the cropping artefacts and the clinical scenario. Fig. S1 depicts the selection criteria and group numbers for ground truth, cropped and filled volumetric data available for analysis. We identified 577 participants in the ADNI dataset having both a T1w and T2w scan at baseline visit. They were divided into three clinical diagnoses: cognitively normal (CN, n = 180; mean age 75 years; mean MMSE score 29; 91 men), mild cognitive impairment (MCI, n = 274; mean age 74; mean MMSE score 27; 168 men), dementia due to probable AD (AD, n = 123; mean age 74; mean MMSE score 23; 65 men). FreeSurfer recon-all segmentation succeeded for all 577 uncropped (ground truth) T1w scans, for 500 cropped T1w scans (137 CN, 245 MCI, 118 AD), and for 560 filled T1w scans (172 CN; 267 MCI; 121 AD). Fig. 3 shows examples of segmentations for original and filled scans where the temporal lobes have been recovered. Table S1 summarises selected demographics of the included ADNI data.


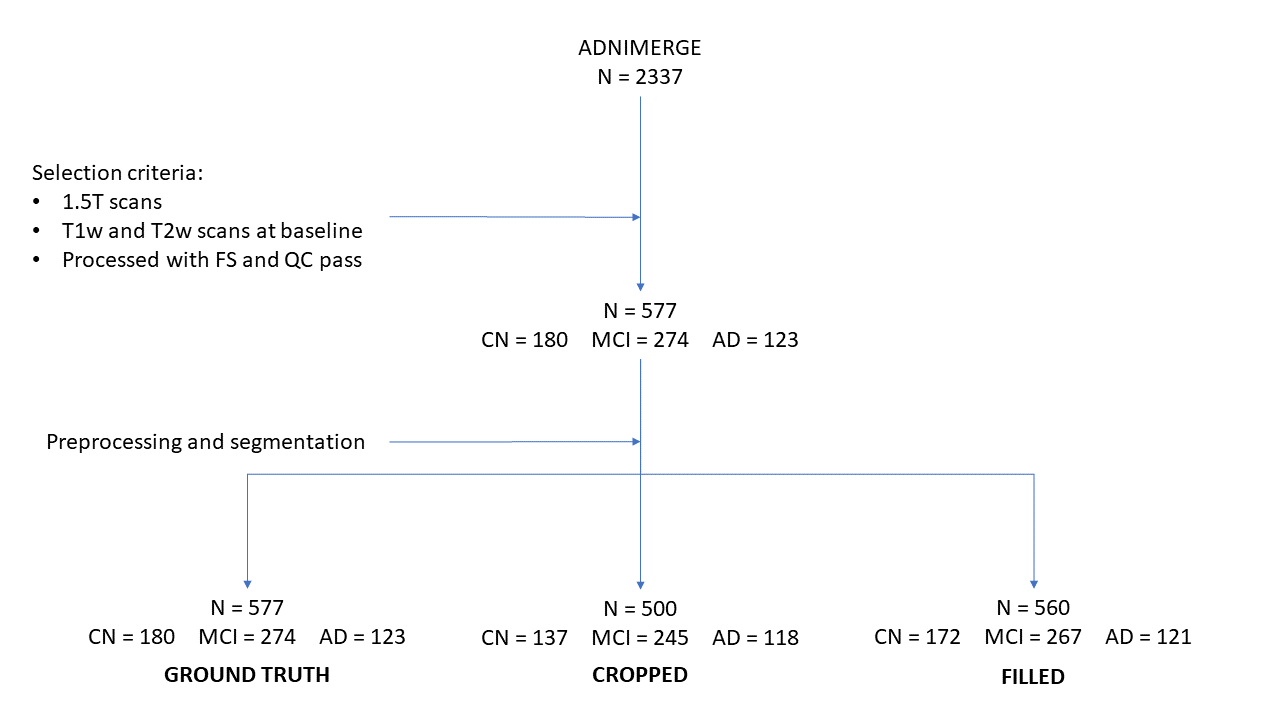


***Fig. S1****: Flow diagram of the study selection process, and final numbers for volumetric measurements of ground truth, cropped and filled scans. FS = FreeSurfer, QC = Quality Check, CN = Cognitively Normal, MCI = Mild Cognitive Impairment, AD = dementia to probable Alzheimer’s Disease.*

***Table S1****: Selected demographics of included ADNI data. Data is presented as mean ± standard deviation.*

|  | **CN** | **MCI** | **AD** |
| --- | --- | --- | --- |
| N, Male (Female) | 172 (89) | 374 (108) | 123 (58) |
| Age, years | 75 ± 5 | 74 ± 7 | 74 ± 8 |
| MMSE | 29 ± 1 | 27 ± 2 | 23 ± 2 |

*Abbreviations: ADNI -- Alzheimer's Disease Neuroimaging Initiative; MMSE -- Mini Mental State Examination; CN -- Cognitively Normal; MCI -- Mild Cognitive Impairment; AD -- dementia due to probable Alzheimer's Disease*

##### MRI Data Collection

The ADNI sample (downloaded in January 2022) included data from all participants who had both a T1w and T2w 3D 1.5T MRI scan at baseline visit from 2005 to 2007 (see Results section). The T1w and T2w scans had resolutions of 1x1x1.25 mm and 1x1x3 mm, respectively. All T1w scans considered for this study had passed a quality check performed by ADNI indicated via a column in the ADNIMERGE table (from <https://ida.loni.usc.edu/pages/access/studyData>). The CODEC sample comprises real-world neuroimaging and clinical data, including neurocognitive test scores. The 3D 1.5T MRI acquisitions are T1w (1x1x1 mm), T2w (0.5x0.5x5 mm) and FLAIR (0.5x0.5x5 to 0.5x0.5x6 mm) scans. The FOV of the T1w sequences is 120x256x224 mm.

##### Image Preprocessing

We pre-processed the ADNI data to resemble the CODEC memory clinic data. This included cropping (artificially zeroing image intensity) the T1w scans outside a lateral FOV of 120 mm and down-sampling the T2w scans to have an axial slice thickness of 5 mm.

##### Imaging Features: regional brain volumes

Estimates of regional brain tissue volumes defined by the Desikan-Killiany atlas^4^ (4) were generated by processing T1w MRI with recon-all (16–18) from FreeSurfer version 7.1.1. This tool involves motion, intensity and bias field correction, skull stripping, volumetric labelling and registration, grey/white matter segmentation and registration to predefined atlases. To reduce dimensionality in the analysis, cortical segmentations were grouped into their respective cortical lobes according to the Klein and Tourville (19) description. Volumetric estimates of cortical and subcortical structures were directly compared between ground truth (uncropped), cropped, and filled T1w MRI.

##### Statistical Analysis

Our crop-filling pipeline was tested by comparing filled and cropped data to uncropped ground-truth data, via regional brain volumes estimated using FreeSurfer. Experiments assessed correlation, bias, statistical differences within and between diagnostic groups, and machine learning classification tasks relevant to Alzheimer’s disease progression. The similarity between regional brain volumes was quantified using Pearson’s correlation coefficient with statistical significance assessed using the two-sided Student t-test. Volumetric correlations were evaluated using the Steiger Z test for measure dependency (20). Biases occurring in the cropped and filled volumetric measurements were analysed using Bland-Altman plots (21) and quantified by mean percentage error.

To investigate whether crop-filling performance varies with disease progression, pipeline performance was assessed using Bonferroni-corrected statistical tests for group differences both within and between diagnostic groups: Cognitively Normal (CN), Mild Cognitive Impairment (MCI), and dementia due to probable Alzheimer’s Disease (AD). This was done using robust linear models that controlled for age, sex, and head size (intracranial volume). A Student’s paired t-test (for Gaussian-distributed data, determined using the Shapiro-Wilk test) or Wilcoxon signed-rank test was used for intra-group comparisons.

Finally, we assessed the influence of cropping (and filling) on data-driven clinical classification tasks relevant to Alzheimer’s disease progression using Support Vector Machines (SVM) (22). For each classification task (CN vs AD, CN vs MCI, MCI vs AD), we used univariate ANOVA to determine which features best discriminated between the respective groups based on F-statistics. The best combination of model hyperparameters (kernel: linear, polynomial, or radial basis function; gamma, C, degree, and nu) was determined using Bayesian optimization. Repeated 10-fold cross-validation was used to evaluate classification performance with training performed on ground truth data and testing on cropped, filled, and ground truth data. Both AUC and balanced accuracy were used as metrics for performance comparison.

##### Results

Partial coverage of the brain showed asymmetric severity, varying across regions of interest and cognitive status, with the left hemisphere affected more than the right hemisphere. The most affected region was the middle temporal (CN: 30/26%, MCI: 29/24.5%, AD: 24.8/21.1%, L/R, other levels of affection are detailed in supplementary Fig. S2).


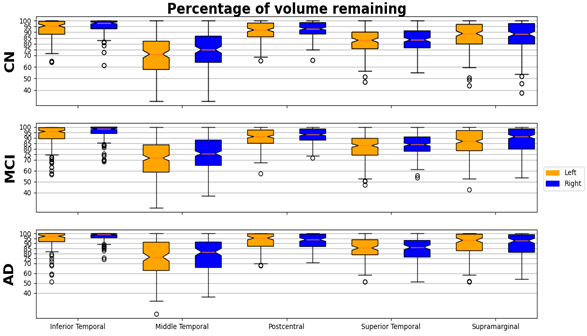


***Fig. S2****: Cropping severity in affected regions. Box plots of the remaining percentage of
volume in cropped T1w sMRI across diagnostic groups. Left hemisphere (orange) and right
hemisphere (blue).*

Correlation and bias results, displayed at Table S4, show that cropping had only marginal effects on subcortical volumes, with crop-filling improving correlation and bias. Table S2 shows results of statistically comparing regional brain volume values in cropped and filled (vs ground truth uncropped) in three key regions of interest for Alzheimer’s disease: ventricles, temporal lobes and hippocampus. All cropped volumes were significantly different for all diagnostic groups. Crop-filling recovered the differences in all regions except the temporal lobe, the most severely affected region due to being the site of cropping.

***Table S2****: P values from group-level comparisons (Student’s t or Wilcoxon signed-rank test) between ground truth (uncropped) volumes and cropped/filled, by diagnosis. Selected regional brain volumes are the same as in Table 2. Note: * denotes P < .0005*

|  | **CROPPED** | | | | **FILLED** | | |
| --- | --- | --- | --- | --- | --- | --- | --- |
|  | **CN** | | **MCI** | **AD** | **CN** | **MCI** | **AD** |
| Lateral Ventricle | |  |  |  |  |  |  |
| Left | | * | * | * | .203 | .915 | .393 |
| Right | | * | * | * | .052 | .504 | .590 |
| Inferior Lateral | |  |  |  |  |  |  |
| Left | | * | * | * | .269 | .918 | .156 |
| Right | | * | * | * | .165 | .921 | .367 |
| Hippocampus | |  |  |  |  |  |  |
| Left | | * | * | * | .896 | .380 | .834 |
| Right | | * | * | * | .021 | .301 | .985 |
| Temporal Lobe | |  |  |  |  |  |  |
| Left | | * | * | * | * | * | * |
| Right | | * | * | * | * | * | * |

Feature selection chose a unique combination of regions (displayed in Fig. S3) for each classification task. In ground truth data, CN and MCI were best separated using volumetric measurements of the left entorhinal cortex and bilateral hippocampus and amygdala. CN and AD were best separated using left inferotemporal, left entorhinal, right middle temporal cortices and bilateral hippocampus and amygdala volumes. MCI and AD were best separated using bilateral inferotemporal, right fusiform cortexes and right hippocampus and amygdala volumes. These findings align well with what is described about the progression of AD according to Braak stages (23): earlier stages (CN vs MCI) are characterised by entorhinal cortex atrophy, followed by the fusiform gyrus and other temporal regions (inferior, middle and superior) in MCI vs AD.


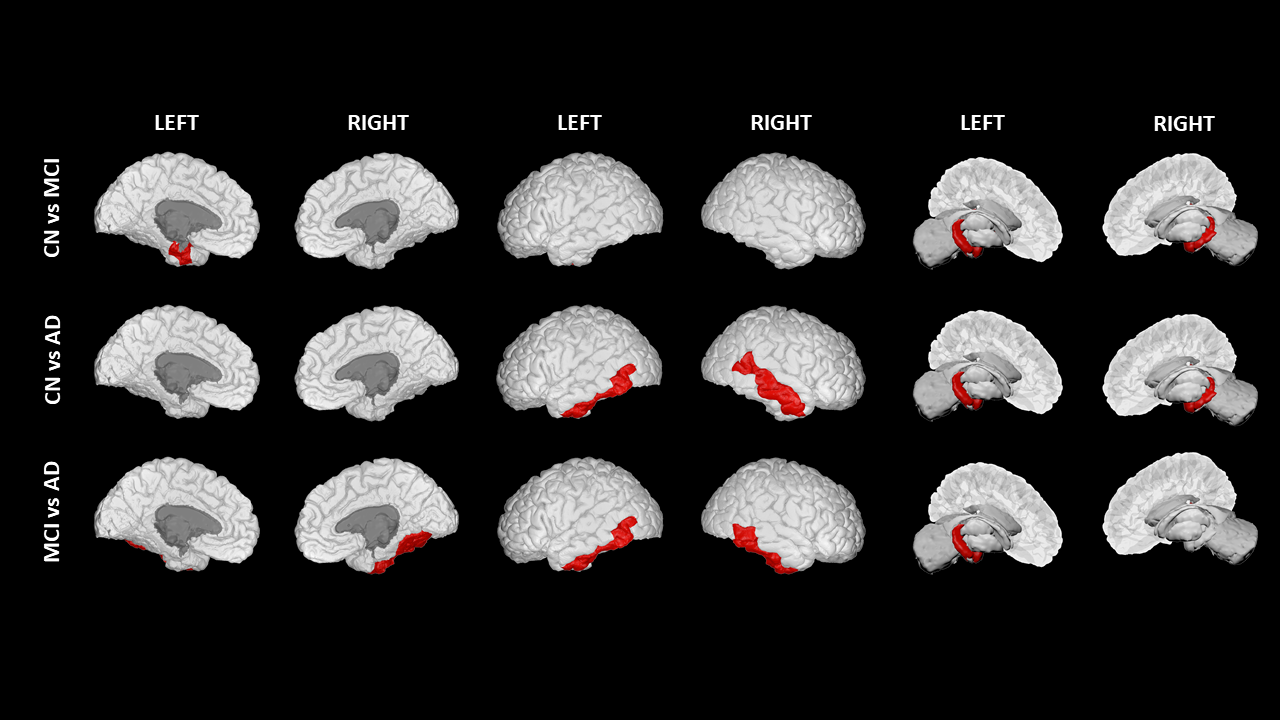


***Fig. S3****: Highlighted ROIs for each classification task. CN = Cognitively Normal, MCI = Mild Cognitive Impairment, AD = dementia to probable Alzheimer’s Disease.*

Table S3 shows AUC and balanced accuracy for the machine learning classification experiments. Results from ground truth data and filled data were statistically indistinguishable in terms of AUC (all p>0.05) and balanced accuracy metrics (all p>0.05). However, when classifying diagnostic groups using cropped data, the model performed similarly to random chance.

***Table S3****: Effects of cropping and filling on AUC and Balanced Accuracy for
each classification task (mean ± standard deviation).*

|  | **AUC** | **BALANCED ACCURACY** |
| --- | --- | --- |
| **CN vs MCI** |  |  |
| Original | 0.80 ± 0.06 | 0.71 ± 0.06 |
| Cropped | 0.54 ± 0.04 | 0.52 ± 0.08 |
| Filled | 0.78 ± 0.06 | 0.70 ± 0.06 |
| **CN vs AD** |  |  |
| Original | 0.93 ± 0.04 | 0.88 ± 0.06 |
| Cropped | 0.55 ± 0.10 | 0.54 ± 0.09 |
| Filled | 0.93 ± 0.05 | 0.87 ± 0.07 |
| **MCI vs AD** |  |  |
| Original | 0.67 ± 0.10 | 0.62 ± 0.07 |
| Cropped | 0.50 ± 0.10 | 0.50 ± 0.06 |
| Filled | 0.67 ± 0.11 | 0.60 ± 0.06 |

Table S4: Correlation (Pearson) and bias (mean percentage difference) of selected regional volumes from cropped and filled scans, relative to ground truth (uncropped). All Pearson correlations were significant with P < .001. Cropped vs filled: * P < .01 (Steiger Z test).

|  |  | **CROPPED** | | **FILLED** | |
| --- | --- | --- | --- | --- | --- |
|  |  | **Pearson r** | **Bias** | **Pearson r** | **Bias** |
| **CN** |  |  |  |  |  |
|  | Lateral Ventricle |  |  |  |  |
|  | Left | >.99 | –.124 | >.99 | –.015 |
|  | Right | >.99 | .124 | >.99 | .015 |
|  | Inferior Lateral |  |  |  |  |
|  | Left | .984 | –1.5 | >.99* | .233 |
|  | Right | .992 | –1.06 | .99 | –.591 |
|  | Hippocampus |  |  |  |  |
|  | Left | .941 | –.141 | .968* | –.351 |
|  | Right | .983 | .413 | .975 | .532 |
|  | Temporal Lobe |  |  |  |  |
|  | Left | .705 | 11.591 | .973* | –3.466 |
|  | Right | .782 | 12.429 | .971* | –1.229 |
| **MCI** |  |  |  |  |  |
|  | Lateral Ventricle |  |  |  |  |
|  | Left | >.99 | .114 | >.99 | .048 |
|  | Right | >.99 | .036 | >.99 | .027 |
|  | Inferior Lateral |  |  |  |  |
|  | Left | >.99* | –.868 | .988 | .484 |
|  | Right | >.99 | .286 | >.99 | .382 |
|  | Hippocampus |  |  |  |  |
|  | Left | .959 | .902 | .952* | –.197 |
|  | Right | .909 | –.805 | .935* | –.3 |
|  | Temporal Lobe |  |  |  |  |
|  | Left | .811 | 1.716 | .969* | –3.827 |
|  | Right | .798 | 12.252 | .976* | –1.594 |
| **AD** |  |  |  |  |  |
|  | Lateral Ventricle |  |  |  |  |
|  | Left | >.99 | .08 | >.99 | –.061 |
|  | Right | >.99 | .13 | >.99 | .047 |
|  | Inferior Lateral |  |  |  |  |
|  | Left | .988 | –.969 | .988 | –1.074 |
|  | Right | >.99 | –.537 | >.99 | –.784 |
|  | Hippocampus |  |  |  |  |
|  | Left | .971 | .499 | .978 | .142 |
|  | Right | .952 | .522 | .954 | .169 |
|  | Temporal Lobe |  |  |  |  |
|  | Left | .869 | 9.292 | .972* | –4.367 |
|  | Right | .887 | 1.354 | .97* | –2.698 |

References:

4. Desikan RS, Ségonne F, Fischl B, et al. An automated labeling system for subdividing the human cerebral cortex on MRI scans into gyral based regions of interest. *NeuroImage*. 2006;31(3):968-980. doi:10.1016/j.neuroimage.2006.01.021

5. Patenaude B, Smith SM, Kennedy DN, Jenkinson M. A Bayesian model of shape and appearance for subcortical brain segmentation. *NeuroImage*. 2011;56(3):907-922. doi:10.1016/j.neuroimage.2011.02.046

6. Lenhart L, Seiler S, Pirpamer L, et al. Anatomically Standardized Detection of MRI Atrophy Patterns in Early-Stage Alzheimer’s Disease. *Brain Sci*. 2021;11(11):1491. doi:10.3390/brainsci11111491

7. Poulakis K, Pereira JB, Mecocci P, et al. Heterogeneous patterns of brain atrophy in Alzheimer’s disease. *Neurobiol Aging*. 2018;65:98-108. doi:10.1016/j.neurobiolaging.2018.01.009

8. Archetti D, Young AL, Oxtoby NP, et al. Inter-Cohort Validation of SuStaIn Model for Alzheimer’s Disease. *Front Big Data*. 2021;4. Accessed May 21, 2023. https://www.frontiersin.org/articles/10.3389/fdata.2021.661110

9. Suppa P, Hampel H, Kepp T, et al. Performance of Hippocampus Volumetry with FSL-FIRST for Prediction of Alzheimer’s Disease Dementia in at Risk Subjects with Amnestic Mild Cognitive Impairment. *J Alzheimers Dis*. 2016;51(3):867-873. doi:10.3233/JAD-150804

10. de Vos F, Schouten TM, Hafkemeijer A, et al. Combining multiple anatomical MRI measures improves Alzheimer’s disease classification. *Hum Brain Mapp*. 2016;37(5):1920-1929. doi:10.1002/hbm.23147

11. Alam S, Kwon GR, Initiative TADN. Alzheimer disease classification using KPCA, LDA, and multi-kernel learning SVM. *Int J Imaging Syst Technol*. 2017;27(2):133-143. doi:10.1002/ima.22217

12. Bartos A, Gregus D, Ibrahim I, Tintěra J. Brain volumes and their ratios in Alzheimer´s disease on magnetic resonance imaging segmented using Freesurfer 6.0. *Psychiatry Res Neuroimaging*. 2019;287:70-74. doi:10.1016/j.pscychresns.2019.01.014

13. Sluimer JD, van der Flier WM, Karas GB, et al. Accelerating regional atrophy rates in the progression from normal aging to Alzheimer’s disease. *Eur Radiol*. 2009;19(12):2826-2833. doi:10.1007/s00330-009-1512-5

14. Dicks E, Vermunt L, van der Flier WM, et al. Modeling grey matter atrophy as a function of time, aging or cognitive decline show different anatomical patterns in Alzheimer’s disease. *NeuroImage Clin*. 2019;22:101786. doi:10.1016/j.nicl.2019.101786

15. Siddiqui TG, Whitfield T, Praharaju SJ, et al. Magnetic Resonance Imaging in Stable Mild Cognitive Impairment, Prodromal Alzheimer’s Disease, and Prodromal Dementia with Lewy Bodies. *Dement Geriatr Cogn Disord*. 2020;49(6):583-588. doi:10.1159/000510951

16. Collins DL, Neelin P, Peters TM, Evans AC. Automatic 3D intersubject registration of MR volumetric data in standardized Talairach space. *J Comput Assist Tomogr*. 1994;18(2):192-205.

17. Dale AM, Fischl B, Sereno MI. Cortical Surface-Based Analysis: I. Segmentation and Surface Reconstruction. *NeuroImage*. 1999;9(2):179-194. doi:10.1006/nimg.1998.0395

18. Fischl B, Dale AM. Measuring the thickness of the human cerebral cortex from magnetic resonance images. *Proc Natl Acad Sci*. 2000;97(20):11050-11055. doi:10.1073/pnas.200033797

19. Klein A, Tourville J. 101 Labeled Brain Images and a Consistent Human Cortical Labeling Protocol. *Front Neurosci*. 2012;6. Accessed July 27, 2023. https://www.frontiersin.org/articles/10.3389/fnins.2012.00171

20. Steiger JH. Tests for comparing elements of a correlation matrix. *Psychol Bull*. 1980;87(2):245-251. doi:10.1037/0033-2909.87.2.245

21. Altman DG, Bland JM. Measurement in Medicine: The Analysis of Method Comparison Studies. *J R Stat Soc Ser Stat*. 1983;32(3):307-317. doi:10.2307/2987937

22. Tanveer M, Richhariya B, Khan RU, et al. Machine Learning Techniques for the Diagnosis of Alzheimer’s Disease: A Review. *ACM Trans Multimed Comput Commun Appl*. 2020;16(1s):30:1-30:35. doi:10.1145/3344998

23. Braak H, Alafuzoff I, Arzberger T, Kretzschmar H, Del Tredici K. Staging of Alzheimer disease-associated neurofibrillary pathology using paraffin sections and immunocytochemistry. *Acta Neuropathol (Berl)*. 2006;112(4):389-404. doi:10.1007/s00401-006-0127-z
